# Autoantibody screening in children who are first-degree relatives of individuals with type 1 diabetes in a sibling-dominant cohort in Turkey: a multicentre study on prevalence, determinants and post-screening parent–child anxiety

**DOI:** 10.1007/s00125-026-06783-6

**Published:** 2026-06-25

**Authors:** Uğur C. Yılmaz, Deniz Ö. Kızılay, Özlem K. Kurt, İlkay B. Balaban Berber, İrem Gökdemir, Aysun Ata, Deniz Erden, Elif G. Basa, Ömer F. Yalçın, Sevim Onguner, Hanife G. Balkı, Sirmen K. Çetin, Samim Özen, Günay Demir, Gamze Y. Baş, Mehmet Soylu, Ahmet Anık, Fatih Gürbüz, Korcan Demir, Özge Köprülü, Selda A. Altıncık, Berna E. Filibeli, Burcu Özbaran, Raika D. Onmuş, Damla Gökşen

**Affiliations:** 1 Department of Pediatric Endocrinology and Diabetes, Ege University Faculty of Medicine, Izmir, Turkey; 2 Department of Pediatric Endocrinology, Adnan Menderes University Faculty of Medicine, Aydın, Turkey; 3 Department of Pediatric Endocrinology, Ankara Bilkent City Hospital, Ankara, Turkey; 4 Department of Pediatric Endocrinology, Afyon Kocatepe University Faculty of Medicine, Afyonkarahisar, Turkey; 5 Department of Pediatric Endocrinology, Dokuz Eylul University Faculty of Medicine, Izmir, Turkey; 6 Department of Pediatric Endocrinology, Dr Behçet Uz Children’s Hospital, Izmir, Turkey; 7 Department of Pediatric Endocrinology, Pamukkale University Faculty of Medicine, Denizli, Turkey; 8 Department of Pediatric Endocrinology, Izmir City Hospital, Izmir, Turkey; 9 Department of Pediatric Endocrinology, Mugla Training and Research Hospital, Mugla, Turkey; 10 Department of Child and Adolescent Psychiatry, Ege University Faculty of Medicine, Izmir, Turkey; 11 Department of Medical Microbiology and Immunology, Ege University Faculty of Medicine, Izmir, Turkey; 12 Department of Public Health, Ege University Faculty of Medicine, Izmir, Turkey

**Keywords:** Anxiety, Autoantibodies, Child, First-degree relatives, Gestational age, Parents, Screening, Siblings, Type 1 diabetes mellitus, Zinc transporter 8

## Abstract

**Aims/objective:**

We screened 2–18-year-old first-degree relatives (FDRs) of individuals with type 1 diabetes in Turkey for diabetes-associated autoantibodies to define their prevalence and age distribution, to identify determinants of autoantibody positivity, and to quantify acute anxiety changes in children and parents after result disclosure.

**Methods:**

In this multicentre cross-sectional study conducted across nine paediatric endocrinology centres in Turkey, 440 FDRs aged 2–18 years were screened. Affected FDR relationships included an affected sister (*n*=206), brother (*n*=204), mother (*n*=22) and father (*n*=13). ZnT8A, GADA, IAA and IA-2A autoantibodies were measured by ELISA; perinatal variables including gestational age were recorded. In the 8–18-year-old subsample, child anxiety was assessed using the full Screen for Child Anxiety Related Emotional Disorders (SCARED) and parental anxiety for these children was assessed using the State–Trait Anxiety Inventory (STAI-I/II), administered before screening and immediately after disclosure.

**Results:**

The proportion of participants who were autoantibody-positive was 16.4% (72/440), and multiple autoantibodies were detected in 13 individuals (3.0%). Among 445 affected FDR relationships, 92.1% were sibling-based and 7.9% were parent-based. ZnT8A was the most common autoantibody (11.4%) and predominated at ages 2–6 years (82% of autoantibody-positive children), declining with age. ZnT8A positivity showed an age-dependent decline, with ZnT8A-positive participants being significantly younger than ZnT8A-negative participants (7.92 ± 4.62 vs 10.00 ± 4.54 years; *p*=0.002). Each additional week of gestational age was associated with lower odds of autoantibody positivity (adjusted OR 0.80 per week; 95% CI 0.68, 0.93; *p*=0.005). This association persisted and was strengthened after exclusion of participants with an affected mother (adjusted OR 0.71 per week; 95% CI 0.59, 0.85; *p*<0.001). SCARED scores increased after disclosure in autoantibody-positive children (*d*=1.31; *p*<0.001) but decreased in autoantibody-negative children (*d*=0.62; *p*<0.001). Parental anxiety increased after positive results (state *d*=2.19; *p*<0.001; trait *d*=0.69; *p*=0.001) and decreased after negative results (*p*<0.001).

**Conclusions/interpretation:**

The ZnT8A predominance in early childhood and the independent association of gestational age with autoantibody positivity support integrating age and perinatal history into presymptomatic risk stratification. These findings should be interpreted primarily within the context of a sibling-dominant paediatric FDR screening cohort. The marked outcome-dependent anxiety response indicates that screening programmes should pair immunological testing with structured, result-specific counselling, integrating psychological support protocols that address differential parent–child anxiety responses with child-appropriate assessment.

**Graphical Abstract:**

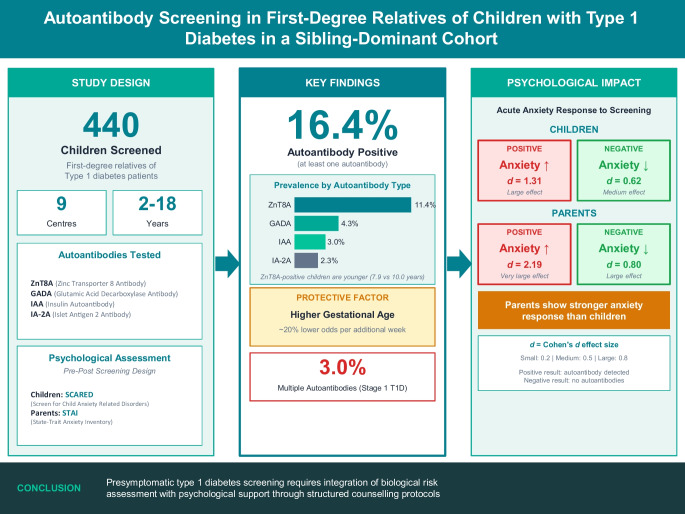

**Supplementary Information:**

The online version contains peer-reviewed but unedited supplementary material available at 10.1007/s00125-026-06783-6.


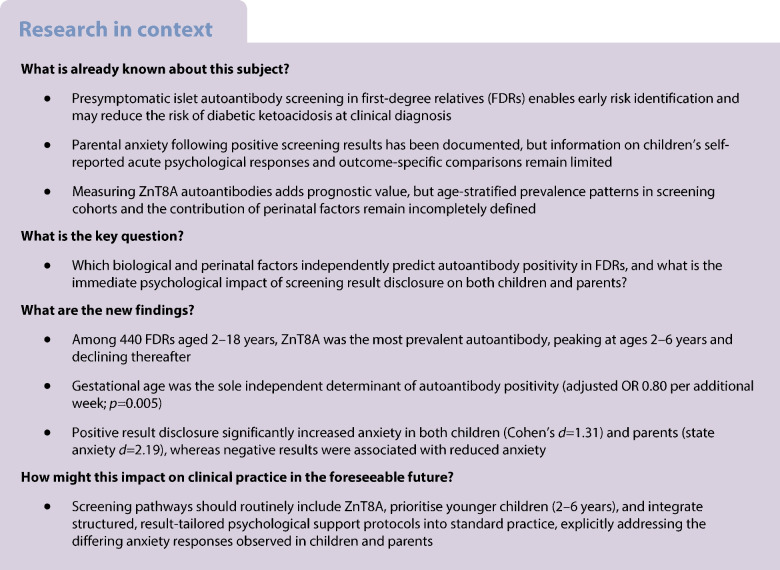


## Introduction

Type 1 diabetes is among the most common chronic metabolic diseases of childhood. Long before clinical diagnosis, a progressive autoimmune process against pancreatic beta cells develops, and is reflected by islet autoantibody positivity [1–3]. In the presymptomatic phase, insulin autoantibody (IAA), glutamic acid decarboxylase antibody (GADA), insulinoma-associated antigen-2 antibody (IA-2A) and zinc transporter 8 antibody (ZnT8A) are among the strongest biomarkers of progression to type 1 diabetes [1–3]. The presence of two or more autoantibodies defines early-stage type 1 diabetes (stages 1–2), and confers a markedly increased short- to mid-term progression risk [1, 3]. Screening enables structured surveillance and may help reduce diabetic ketoacidosis (DKA) risk and preserve residual C-peptide through timely diagnosis [4].

In Turkey, where DKA rates at diagnosis remain high (approximately 30–40%) [5–8], the case for presymptomatic screening is compelling. Cost-effectiveness analyses support screening in high-DKA settings [9, 10], and TrialNet and Fr1da study data show that early identification reduces DKA [11–13]. In parallel, teplizumab offers a disease-modifying option to delay progression [14]. Although general population screening offers broader coverage because approximately 90% of new type 1 diabetes cases occur in families without an affected first-degree relative (FDR), targeted FDR screening remains pragmatic, yielding an approximately fourfold higher capture rate for preclinical stage 1–2 type 1 diabetes than general population screening (1.07% vs 0.29%) [15, 16]. Given that FDRs have an approximately 15-fold higher type 1 diabetes risk than the general population [1, 13, 17], screening in this group may reduce symptom burden, prevent DKA, and facilitate timely counselling.

Current guidance recommends confirmatory testing, structured glycaemic monitoring, family counselling and referral to specialist centres for individuals identified as having presymptomatic type 1 diabetes [4, 13, 18]. The psychological impact of screening requires systematic consideration: ISPAD consensus guidance emphasises evaluation and management of psychosocial effects within structured counselling and follow-up frameworks [13, 19], and early anxiety responses in population-based programmes may attenuate over time with ongoing follow-up [20]. However, this pattern may vary by risk level, with parental anxiety remaining elevated over time among parents of children with multiple islet autoantibodies, but decreasing in lower-risk groups [21]. Psychosocial evidence has primarily focused on parental anxiety, and data on children’s anxiety are limited: most studies have used short State–Trait Anxiety Inventory (STAI) forms in parents [20, 21], but comprehensive child instruments across broader age ranges and anxiety domains (such as the full Screen for Child Anxiety Related Emotional Disorders; SCARED) have not been systematically applied in presymptomatic type 1 diabetes screening.

To address this gap, this multicentre study in Turkey simultaneously evaluated islet autoantibody screening and its psychological impact in FDRs of children and adolescents with type 1 diabetes, and is among the few to have administered the full SCARED questionnaire in children alongside the full STAI-I/II questionnaires in parents. By integrating biological and psychosocial measures in a high-DKA context, we aimed to inform screening algorithms that are both effective for risk detection and psychologically safe for clinical implementation.

## Methods

### Study design and centres

**Fig. 1:**
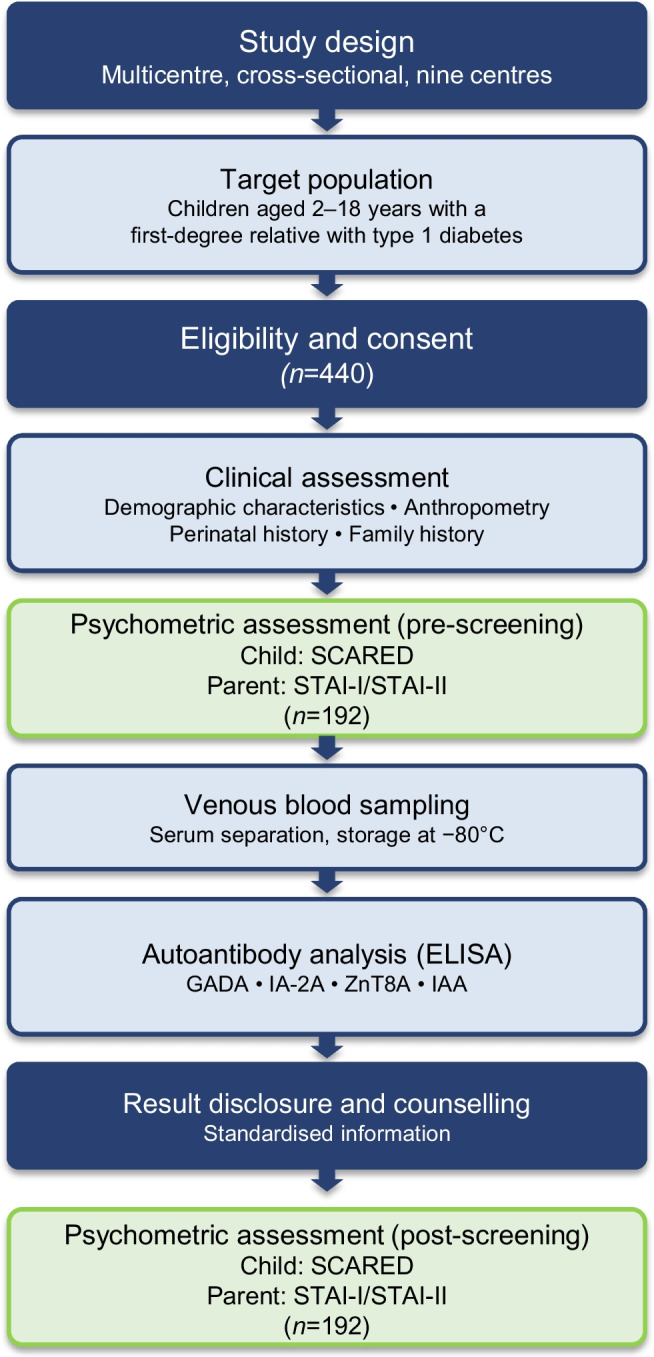
Study flow diagram summarising workflow of this multicentre cross-sectional screening study conducted across nine paediatric endocrinology centres in Turkey. Children aged 2–18 years with an FDR with type 1 diabetes were consecutively invited; eligible participants provided consent/assent and were enrolled (*n*=440). Baseline assessment included demographic characteristics, anthropometry, perinatal history and family history. Before screening, children completed SCARED and parents completed STAI-I/II. Venous blood was collected, serum was separated and stored at −80°C, and islet autoantibodies (GADA, IA-2A, ZnT8A and IAA) were measured by ELISA. Results were disclosed with standardised counselling, and SCARED and STAI-I/II were repeated 15–30 min after disclosure/counselling to assess the acute psychological responses

This multicentre cross-sectional screening study with short-term follow-up was conducted between June 2024 and June 2025, and coordinated by Ege University Faculty of Medicine. A total of nine university hospitals participated in the study, representing the seven geographical regions of Turkey. Data collection, sampling, laboratory workflows and anxiety scale administration were standardised across centres using a common protocol. Eligible FDRs were invited in line with risk-based screening strategies [13]. The workflow is summarised in Fig. 1.

### Participants

We enrolled 440 non-diabetic children/adolescents (2–18 years) with at least one parent or sibling with type 1 diabetes. Sample size calculation assumed approximately 12% autoantibody positivity (95% confidence, 80% power). Participants were recruited consecutively from the paediatric endocrinology clinics of nine participating university hospitals located across various geographical regions of Turkey. Race and ethnicity data were not collected, as ethnicity is not routinely recorded in Turkish demographic or healthcare datasets; neither were individual-level socioeconomic data systematically collected. Inclusion criteria were age 2–18 years, an affected biological parent/sibling, and provision of written informed consent from the parents/legal guardians, with written and/or verbal assent being obtained from children aged ≥12 years, in accordance with the protocol approved by the institutional ethics committee. Exclusion criteria included diabetes; hyperglycaemia symptoms or incidental hyperglycaemia; monogenic diabetes or pancreatectomy; autoimmune polyglandular syndrome or immunodeficiency; recent immunosuppressive/immunomodulatory therapy (≤6 months); inability to complete psychometric scales; or insufficient/missing data.

### Data collection and clinical variables

Age, sex and kinship to the affected FDR (mother, father, sister, brother) were recorded using a standardised case report form. Sex was recorded from clinical records and/or parent-reported information at enrolment; gender identity was not systematically collected. Five participants had both an affected sibling and an affected parent (two mother + sibling, three father + sibling). Height, weight and BMI SD scores were calculated using Turkish reference charts [22]. Mode of delivery and gestational age were recorded; gestational age was analysed as both a continuous variable and a categorical variable (preterm, early term, full term, post-term). Venous blood was collected for autoantibody testing: information on autoantibody status (positive/negative), type, number and multiple positivity (two or more) was derived from the results, and the data were entered into an electronic database.

### Autoantibody measurements

Centrally analysed serum samples (stored at −80°C) were tested for islet autoantibodies by ELISA (GADA, IA-2A and ZnT8A supplied by Euroimmun, Lübeck, Germany; IAA supplied by Orgentec Diagnostika, Mainz, Germany). Positivity cut-offs were >10 IU/ml (GADA, IA-2A and IAA) and >15 RU/ml (ZnT8A). Having two or more positive autoantibodies defined presymptomatic stage 1 type 1 diabetes [13].

### Psychometric assessment

Child anxiety was assessed using SCARED [23, 24], a 41-item self-report instrument with five subscales (separation anxiety, generalised anxiety, social phobia, panic/somatic symptoms, and school avoidance). SCARED has established Turkish validity and reliability [24] across a broad paediatric age range, and was selected to allow a uniform assessment in participants aged 8–18 years. In this study, SCARED was used to capture children’s overall anxiety symptom burden across multiple domains rather than reflecting a momentary state-anxiety response. Although child-specific state-anxiety instruments are available, validation data across a similarly broad paediatric age range are more limited; therefore, SCARED was preferred for consistent application within this cohort. In line with the validated age range of the instrument, SCARED was administered only to participants aged 8–18 years. Of 283 eligible participants, 192 provided complete SCARED questionnaires for psychometric analyses; 91 of the original 283 were excluded because they did not participate in the psychometric assessment or returned questionnaires with missing responses that precluded calculation of SCARED total and/or subscale scores. Parental anxiety was assessed using STAI-I/II [25, 26]. STAI-I evaluates state anxiety, reflecting the acute emotional response to a stressor at a given moment, whereas STAI-II evaluates trait anxiety, reflecting a more stable tendency to experience anxiety. Thus, these tools assess related but distinct constructs. For each child, one parent (preferably the primary caregiver) completed the STAI-I/II assessments. The final parent STAI-I/II analytic sample comprised 192 parents; forms with missing responses that precluded calculation of STAI-I or STAI-II scores were not included. Significant anxiety was defined as a SCARED score ≥25 and STAI-I or STAI-II scores ≥40 [23–26]. Assessments followed a harmonised protocol by trained investigators. Participants with clinically significant scores were referred for further evaluation and support.

### Result disclosure, counselling and post-disclosure assessment

Results were disclosed using an ISPAD-aligned script [13]. Counselling included discussion of the interpretation of positive and negative results, individualised progression risks, symptom education and follow-up plans. Families with negative results were advised of a residual risk and offered annual re-screening [4]. Participants with one or more autoantibody, and particularly those with multiple positivity (two or more autoantibodies), were referred for paediatric endocrinology follow-up and structured glycaemic monitoring/periodic dysglycaemia assessment as appropriate [13].

Completion of SCARED (children) and STAI-I/II (parents) was repeated 15–30 min after disclosure/counselling to assess the acute psychological response; invalid reassessments were excluded.

### Statistical analysis

Analyses were performed using SPSS Statistics version 27 (IBM, Armonk, NY, USA). The results for continuous variables are presented as mean ± SD. Independent-group comparisons were performed using unpaired Student's *t* tests or Mann–Whitney *U* tests, with the parametric or non-parametric test being selected based on visual inspection of histograms and Q–Q plots for approximate normality; categorical variables were compared using a χ^2^ test or Fisher’s exact test, as appropriate. Autoantibody positivity was examined using multivariable logistic regression. For psychological outcomes, a pre-specified objective was to assess acute within-participant change after result disclosure using paired comparisons and change-score modelling. Pre- and post-disclosure scores were compared using paired Student’s *t* tests. Exploratory paired analyses of SCARED subscales were performed in the autoantibody-positive subgroup. In antibody-negative sibling FDR participants, an exploratory post hoc mixed-design ANOVA was used to examine the effects of time, child sex and affected-sibling sex on child anxiety scores. Change in child anxiety was additionally quantified as ΔSCARED, defined as the total SCARED score post-disclosure minus the total SCARED score pre-disclosure, and analysed using multivariable linear regression, with the pre-disclosure SCARED score and autoantibody status entered as predictors. Parental STAI outcomes were evaluated using paired comparisons and effect size. No formal adjustment for multiple comparisons was applied; SCARED subscale analyses and post hoc subgroup analyses are reported as exploratory and interpreted accordingly. Two-sided *p* values <0.05 were considered significant.

### Ethics approval

The Ege University Medical Research Ethics Committee approved this multicentre study (no: 24-4T/74, 4 April 2024). The study was conducted according to Helsinki and ICH good clinical practice guidelines; written parental consent was obtained for all participants and assent was obtained from children aged ≥12 years, ensuring data confidentiality.

## Results

A total of 440 FDRs were enrolled (47.7% male; mean age 9.77 ± 4.59 years; mean gestational age 38.44 ± 1.43 weeks). Among the 445 affected FDR relationships reported in these 440 participants, 92.1% were sibling-based and 7.9% were parent-based. Perinatal, anthropometric and kinship characteristics are summarised in Table 1.

**Table 1:** Demographic and clinical characteristics according to islet autoantibody status, with bivariate and multivariable analyses

Variable	Total (*n*=440)	Autoantibody-negative (*n*=368)	Autoantibody-positive (*n*=72)	Bivariate *p* value	aOR	95% CI	Adjusted *p* value
Age (years)	9.77 ± 4.59	9.90 ± 4.54	9.05 ± 4.96	0.129	0.96	0.91, 1.02	0.173
Sex^a^				0.166	1.40	0.83, 2.37	0.211
Female	230 (52.3)	187 (50.8)	43 (59.7)				
Male	210 (47.7)	181 (49.2)	29 (40.3)				
Mode of delivery^b^				0.930	0.84	0.48, 1.45	0.520
Vaginal	163 (37.0)	136 (37.0)	27 (37.5)				
Caesarean	277 (63.0)	232 (63.0)	45 (62.5)				
Gestational age (weeks)	38.44 ± 1.43	38.53 ± 1.47	37.94 ± 1.09	<0.001	0.79	0.67, 0.92	0.003
Breastfeeding duration (months)	14.61 ± 6.90	14.43 ± 6.77	15.89 ± 7.53	0.079	1.03	0.99, 1.07	0.155
Weight SD score	0.22 ± 1.37	0.24 ± 1.38	0.09 ± 1.23	0.382	–	–	–
Height SD score	0.13 ± 1.21	0.15 ± 1.23	−0.02 ± 0.94	0.302	–	–	–
BMI SD score	0.16 ± 1.34	0.17 ± 1.36	0.04 ± 1.34	0.433	–	–	–
BMI (kg/m^2^)	19.15 ± 4.53	19.19 ± 4.54	18.43 ± 3.99	0.218	–	–	–
FDR with T1D^c^				0.196	1.61	0.95, 2.71	0.074
Sister	206 (46.3)	165 (44.2)	41 (56.9)				
Brother	204 (45.8)	179 (48.0)	25 (34.7)				
Mother	22 (4.9)	18 (4.8)	4 (5.6)				
Father	13 (2.9)	11 (2.9)	2 (2.8)				
Sibling sex (sister vs brother)^d^	41/206 vs 25/204	–	–	0.043	–	–	–

Data are presented as mean ± SD for continuous variables or *n* (%) for categorical variables. Bivariate *p* values were calculated using Student's *t* test or the Mann–Whitney *U* test (for continuous variables) or using the χ^2^ test or Fisher's exact test (for categorical variables). The multivariable logistic regression model included age, sex, gestational age, breastfeeding duration, mode of delivery and family history of T1D in a sister (*n*=440; Nagelkerke pseudo-*R*^2^=0.047). Anthropometric variables were not included in the pre-specified multivariable model, and are presented for descriptive purposes only. Five participants had more than one affected FDR; therefore, the FDR categories correspond to 445 relationships across 440 participants. A *p* value <0.05 was considered statistically significant

^a^Female was used as the reference

^b^Vaginal delivery was used as the reference

ᶜPresence of an affected sister vs all other kinship categories (participant-level coding in the multivariable model)

^d^The 'sibling sex' comparison shows the proportion of autoantibody-positive individuals among siblings, stratified by the sex of the affected sibling (41/206 [19.9%] for those with an affected sister vs 25/204 [12.3%] for those with an affected brother)

T1D, type 1 diabetes

**Fig. 2:**
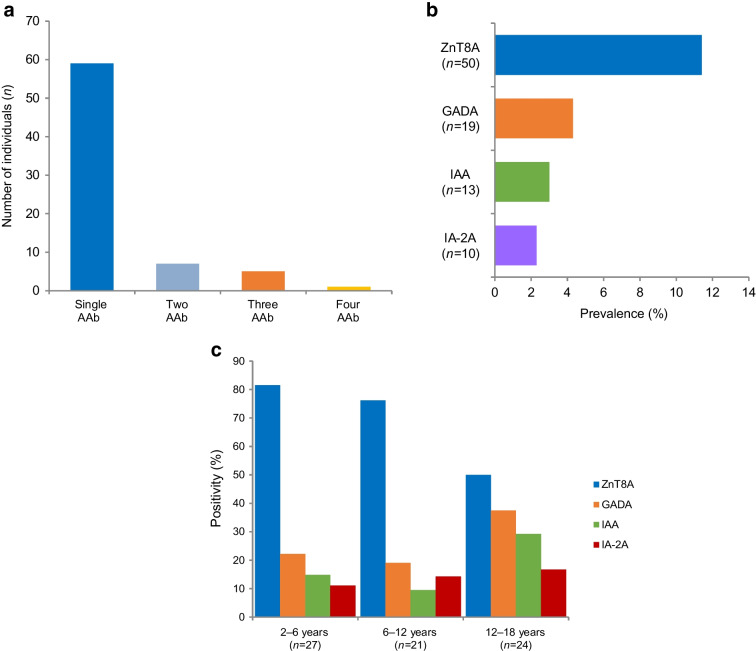
Islet autoantibody prevalence, distribution and age-related patterns. (**a**) Number of positive islet autoantibodies among screened FDRs (*n*=440). (**b**) Prevalence of individual autoantibodies in the total cohort (*n*=440). Participants could be positive for more than one autoantibody. (**c**) Autoantibody patterns by age group among autoantibody-positive participants (*n*=72). ZnT8A positivity decreased with age (*p*=0.037 χ^2^ test); no significant age group differences were observed for GADA (χ^2^ test), IAA (Fisher's exact test) or IA-2A (Fisher's exact test). The *p* values reflect comparisons across age groups (χ^2^ or Fisher's exact test, as appropriate). AAb, autoantibody/autoantibodies

Islet autoantibodies were detected in 16.4% of participants (*n*=72). Antibody positivity was single in 59 of these participants (13.4% of the total) and multiple (two or more) in 13 (3.0% of the total). Two antibodies in were detected in seven participants (1.6%), three antibodies were detected in five participants (1.1%), and four antibodies were detected in one participant (0.2%). Specific autoantibody rates were 11.4% for ZnT8A (*n*=50), 4.3% for GADA (*n*=19), 3.0% for IAA (*n*=13) and 2.3% for IA-2A (*n*=10) (Fig. 2).

Autoantibody-positive and -negative participants significantly differed only in gestational age (37.94 ± 1.09 vs 38.53 ± 1.47 weeks; *p*<0.001) (Table 1). The proportion of autoantibody positivity was higher among participants with an affected sister than among those with an affected brother (19.9% vs 12.3%, *p*=0.043).

ZnT8A-positive participants were significantly younger than ZnT8A-negative participants (7.92 ± 4.62 vs 10.00 ± 4.54 years; *p*=0.002), consistent with the observed age-dependent decline in ZnT8A positivity. Among autoantibody-positive participants, ZnT8A positivity decreased across age groups from 81.5% in those aged 2–6 years, to 76.2% in those aged 6–12 years, and 50.0% in those aged 12–18 years (*p*=0.037). Logistic regression confirmed that ZnT8A was the only autoantibody whose presence was inversely associated with age (*p*=0.005); no significant age-related differences were found for GADA, IAA or IA-2A (Fig. 2).

After adjustment, only gestational age remained independently associated with autoantibody positivity; having an affected sister was not significantly associated with this outcome (aOR=1.61; 95% CI 0.95, 2.71; *p*=0.074). In the core model (age, sex, family history), higher gestational age was associated with a lower adjusted OR (aOR=0.80; 95% CI 0.68, 0.93; *p*=0.005), and the association remained robust after additional adjustment for breastfeeding duration and delivery mode (aOR=0.79; 95% CI 0.67, 0.92; *p*=0.003), corresponding to an approximately 20% reduction in odds per additional week of gestation. To assess whether this association is influenced by the small maternal FDR subgroup, we performed a sensitivity analysis that excluded participants with an affected mother; gestational age remained independently associated with autoantibody positivity (aOR=0.71; 95% CI 0.59, 0.85; *p*<0.001).

The autoantibody positivity rates across the gestational age categories (preterm, early term, full term and post-term) were 14.3%, 43.3%, 3.5% and 0%, respectively. Positivity was highest in the early-term group and lower in the later gestational age categories; overall, the Cochran–Armitage trend test showed a significant inverse trend (*p*<0.001). ZnT8A and IAA were moderately negatively correlated (*r*=−0.55; *p*<0.001), whereas GADA and IA-2A were positively correlated (*r*=0.31; *p*=0.009).

### Changes in anxiety symptoms in children and parents

**Fig. 3:**
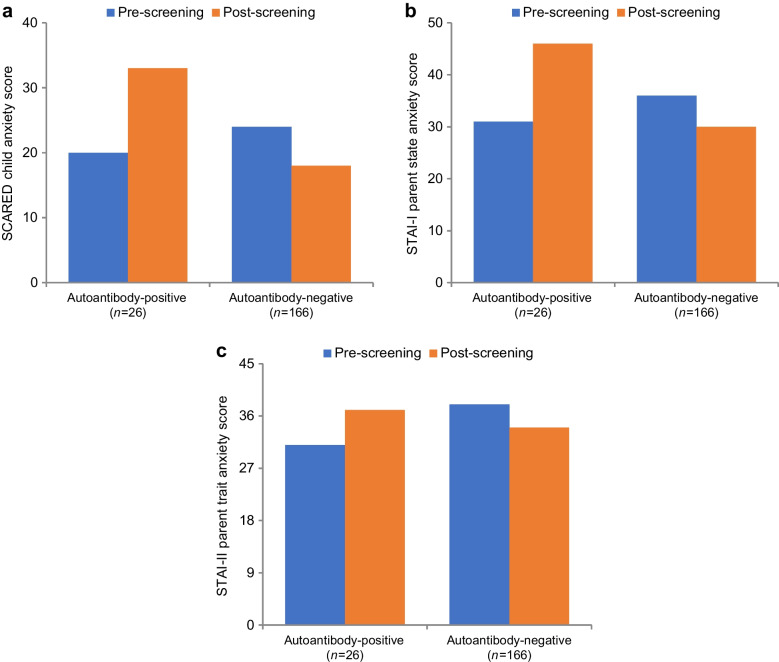
Impact of autoantibody screening results on child and parent anxiety levels. Pre- and post-disclosure anxiety scores are shown for participants with complete paired assessments (*n*=192), stratified by screening result (autoantibody-negative vs autoantibody-positive). (**a**) Child anxiety scores (SCARED). (**b**) Parent state-anxiety scores (STAI-I). (**c**) Parent trait anxiety scores (STAI-II). Raw pre- and post-disclosure scores are shown; change scores (post-disclosure minus pre-disclosure) are reported in the text and ESM Tables 2, 6 and 7

The SCARED analyses included 192 children/adolescents (56% female, *n*=108; 44% male; *n*=84); 26 of these participants (14%) were autoantibody-positive and 166 (86%) autoantibody-negative. Their mean age was 12.32 ± 2.99 years (range 8–18) (see electronic supplementary material [ESM] Table 1). The total SCARED score decreased from 23.38 ± 12.23 pre-screening to 19.89 ± 10.83 post-screening (mean difference −3.49 ± 11.60; *p*<0.001), corresponding to a small effect size (Cohen’s *d*=0.30; 95% CI 0.16, 0.44) (Fig. 3). The SCARED scores decreased in both girls (*p*=0.008) and boys (*p*=0.002), and SCARED change scores were not correlated with age (ESM Table 2).

The SCARED score increased in autoantibody-positive participants from 20.15 ± 10.47 to 32.88 ± 13.83 (mean change 12.73 ± 9.68; *p*<0.001; *d*=1.31; 95% CI 0.80, 1.80) but decreased in autoantibody-negative participants from 23.88 ± 12.43 to 17.85 ± 8.72 (mean change −6.03 ± 9.68; *p*<0.001; *d*=0.62; 95% CI 0.45, 0.79). Exploratory paired-samples subscale analyses were performed within the autoantibody-positive subgroup, and it was found that the increase in total SCARED scores was primarily driven by increases in panic/somatic symptoms (from 4.27 ± 2.18 to 14.12 ± 5.47; *p*<0.001) and in the generalised anxiety subscale (from 3.27 ± 1.69 to 10.27 ± 4.07; *p*<0.001) in the autoantibody-positive group (ESM Table 3), whereas both subscales decreased in the autoantibody-negative group (from 5.73 ± 3.25 to 4.95 ± 3.06; *p*=0.01 and from 4.57 ± 2.77 to 3.90 ± 2.22; *p*=0.005, respectively) (ESM Table 4). Similar patterns were observed for separation anxiety, social anxiety and school avoidance/phobia in both groups (ESM Tables 3 and 4). In multivariable linear regression, ΔSCARED (post-disclosure score minus pre-disclosure score) was independently associated with the baseline SCARED score (β=−0.51; *p*<0.001) and autoantibody positivity (β=16.87; *p*<0.001) (*R*^2^=0.591; adjusted *R*^2^=0.587; *p*<0.001) (ESM Table 5).

In an exploratory post hoc analysis of autoantibody-negative sibling FDRs, mixed-design ANOVA showed a main effect of time on child anxiety (*F*(1,162)=63.66, *p*<0.001, partial η^2^=0.282), with lower scores after disclosure. No significant effects were found for child sex, affected-sibling sex or their interactions (ESM Table 6).

Among the corresponding 192 parents, the state-anxiety score (STAI-I) decreased from 35.10 ± 8.60 pre-screening to 32.22 ± 8.89 post-screening (mean difference −2.88 ± 10.27; *p*<0.001) and the trait anxiety score (STAI-II) decreased from 36.67 ± 8.45 to 34.83 ± 8.33 (mean difference −1.83 ± 7.16; *p*<0.001) (STAI-I *d*=0.28; 95% CI 0.14, 0.43; STAI-II *d*=0.26; 95% CI 0.12, 0.41).

Among parents of autoantibody-positive children (*n*=26), the STAI-I score increased (from 30.67 ± 8.18 to 45.78 ± 7.46; mean change +15.11 ± 6.89; *p*<0.001) as did the STAI-II score (from 31.26 ± 5.91 to 37.19 ± 8.84; mean change +5.93 ± 8.59; *p*=0.001). Among parents of autoantibody-negative children (*n*=166), the STAI-I score decreased (35.83 ± 8.47 to 30.01 ± 6.94; mean change −5.82 ± 7.31; *p*<0.001) as did the STAI-II score (37.55 ± 8.49 to 34.45 ± 8.20; mean change −3.10 ± 6.04; *p*<0.001). The effect sizes were *d*=2.19 (95% CI 1.46, 2.92) and *d*=0.69 (95% CI 0.26, 1.12), respectively, in the autoantibody-positive group, and *d*=0.80 (95% CI 0.61, 0.99) and *d*=0.51 (95% CI 0.33, 0.69), respectively, in the autoantibody-negative group (Fig. 3; ESM Table 7). To facilitate subgroup comparisons, Cohen’s *d* effect size with 95% CI for within-group changes in the SCARED and STAI-I/II scores are summarised in ESM Fig. 1.

## Discussion

This large-scale screening study in Turkey concurrently evaluated perinatal characteristics, islet autoantibodies, and changes in anxiety symptoms in FDRs of children with type 1 diabetes. Our findings suggest that presymptomatic type 1 diabetes screening should combine biological risk stratification with safeguards for the psychological safety of parents and children. The inverse association between ZnT8A positivity and age, together with gestational age as an independent determinant of autoantibody positivity, provides actionable evidence on whom and when to screen, and how to refine risk profiles.

An important feature of our cohort is its marked sibling predominance: among 445 recorded affected FDR relationships, 410 (92.1%) were sibling-based and 35 (7.9%) were parent-based. Accordingly, the present study should be interpreted primarily within the context of a sibling-dominant paediatric FDR screening study rather than as an evenly distributed parent–sibling FDR cohort. We therefore consider the parental FDR observations secondary and exploratory; the main inferences of the study relate to children with an affected sibling.

### Prevalence and comparison with the literature

Our finding of 16.4% (72/440) islet autoantibody positivity in FDRs is broadly consistent with the 10–15% ISPAD guideline [13] and the results from prior high-risk cohorts (e.g. TEDDY, TrialNet) [2, 3, 11, 17, 22, 27–29]. In contrast, population screening in the Fr1da study reported 0.31% positivity in the general childhood population [16], whereas the TrialNet Pathway to Prevention study demonstrated substantially higher detection rates among FDRs [11]. The prevalence in the present study (slightly above the 10–15% ISPAD reference range for FDR cohorts [13]) may reflect context-specific factors that compound genetic susceptibility in this population.

Targeted screening yields higher detection than general population screening [11, 16, 28, 29], supporting careful risk group selection in resource-limited settings. As the first multicentre study reporting islet autoantibody prevalence in FDRs of children with type 1 diabetes in Turkey, these findings may guide national screening strategies and also provide reference data for international comparisons.

### Multiple autoantibody positivity

Multiple autoantibody positivity (two or more autoantibodies) was identified in 3.0% of participants, consistent with the natural history of presymptomatic type 1 diabetes. The results from TEDDY and other cohorts show that multiple positivity is associated with a substantially higher progression risk than single positivity and reflects an advanced autoimmune stage [2, 3, 17, 22, 27]. In our cohort, multiple positivity involving progression-linked antibodies (e.g. ZnT8A and/or IA-2A) occurred at younger ages than single positivity, highlighting early childhood as a critical window for rapid autoimmunity. Despite its low prevalence, multiple positivity identifies a higher-risk subgroup that warrants closer metabolic monitoring, including more frequent assessment for dysglycaemia, in line with ISPAD recommendations [13].

### ZnT8A positivity and its relationship with age

ZnT8A was the most frequent autoantibody (11.4%), and its prevalence declined significantly with age (*p*=0.037), from an early childhood peak. This contrasts with IAA-dominant birth cohorts such as TEDDY [2] and GADA-dominant FDR screens [28], highlighting that autoantibody hierarchy varies strongly by population, ethnicity and context [30, 31].

Evidence suggests that ZnT8A often appears early in seroconversion, commonly alongside other islet autoantibodies, and its presence may capture early presymptomatic type 1 diabetes [32, 33]. Together with the age-dependent distribution observed here, our findings support a distinct role for ZnT8A in immunological staging, and reinforce recommendations that ZnT8A be included in screening panels, particularly in younger age groups [13]. Incorporating age-specific ZnT8A patterns into risk models may further refine staging and improve early phenotype detection.

### Gestational age and autoantibody positivity

The inverse association between gestational age and islet autoantibody positivity is a novel finding of this study, and has previously been supported only indirectly [30, 31, 34]. Preterm and late-preterm birth may have lasting effects on immune development (immune tolerance, regulatory T cell maturation, epigenetic programming, and neonatal microbiota colonisation) [30, 34]; immune maturation in preterm infants has been reported to differ structurally and functionally from that in term infants [35]. Consistent with this biological framework, large Nordic cohorts have shown higher type 1 diabetes risk after preterm birth, including a Swedish cohort of more than 4 million individuals (21–24% increased risk) [36] and a 5.5 million person register study from Finland/Norway/Sweden, with the highest risk at gestational week 37 [37]. In our study, each additional week of gestation was associated with an approximately 20% reduction in the odds of autoantibody positivity, suggesting that gestational age may be a biological determinant of presymptomatic type 1 diabetes. Mechanistic studies (e.g. on T cell maturation, epigenetic signatures and microbiota profiles) are warranted, and incorporating gestational age into risk models may support closer follow-up, earlier counselling and tailored risk communication for those born before term.

Although the number of perinatal variables assessed was limited, gestational age remained independently associated with autoantibody positivity in a sensitivity analysis that excluded participants with an affected mother, with a slightly stronger effect than in the full cohort.

### Intrafamilial risk patterns

The borderline association between having an affected sister and islet autoantibody positivity (aOR=1.61; *p*=0.074) is hypothesis-generating, and may suggest sex-specific intrafamilial risk patterns. Risk among FDRs reflects not only shared HLA background and genetic susceptibility [38] but also intrafamilial environmental factors, pregnancy history, and parent–child interactions and sibling interactions [39]. Given the sample size and borderline statistical significance, this finding should be interpreted as a preliminary observation requiring confirmation in larger, prospective cohorts; nonetheless, it provides a rationale for future studies on sex-specific intrafamilial risk, an area that remains under-studied.

### Impact on anxiety symptoms in children

Our findings address an important gap regarding children’s anxiety responses to presymptomatic type 1 diabetes screening. Most prior studies have focused on parental anxiety and risk perception, whereas children’s own anxiety has often been unassessed [20, 21, 40], particularly in the presymptomatic screening phase. In our study, use of the full SCARED questionnaire allowed a standardised assessment of anxiety symptoms in children aged 8–18 years [23, 24]. The reduction in total SCARED scores in the overall sample after screening suggests that structured information and counselling may reduce uncertainty and future-oriented anxiety in some children. In contrast, SCARED scores increased markedly among those receiving an autoantibody-positive result (*d*=1.31), indicating a strong and clinically meaningful acute emotional response to risk disclosure. Because SCARED was not designed to capture immediate state-related anxiety changes around a single stressor, this finding should be interpreted cautiously. Exploratory subscale analyses suggested that the increase in total SCARED scores among autoantibody-positive children was driven primarily by panic/somatic and generalised anxiety symptoms, rather than representing a uniform rise across all anxiety domains. This pattern may indicate that disclosure of a positive islet autoantibody result evokes acute internal distress and somatic apprehension rather than broader anxiety responses. As one of the first large-scale studies to quantify child anxiety in presymptomatic type 1 diabetes screening using SCARED, our findings support integrating child-centred self-report measures into screening pathways, consistent with contemporary psychosocial care guidelines [19]. Although anxiety was assessed only around result disclosure, its longer-term trajectory warrants further study.

Among antibody-negative sibling FDR participants, the post-disclosure decline in anxiety did not differ by child sex or affected-sibling sex, suggesting reassurance from a negative result rather than sex-specific sibling effects. However, the study was not powered for sex- or gender-stratified analyses, and gender identity was not systematically collected; therefore, results for sex- or gender-specific effects should be interpreted cautiously.

### Impact on anxiety symptoms in parents

Changes in parental state and trait anxiety showed a bidirectional pattern following screening. In the overall sample, post-screening reductions in the STAI-I and STAI-II scores suggest that a well-structured screening process may reduce uncertainty. Previous studies, mostly using abbreviated instruments rather than the full STAI-I/II questionnaires, have suggested that parental anxiety responses differ by risk level, tending to remain elevated in higher-risk groups and to decline in lower-risk groups [20, 21]. In contrast, in our study, parents receiving an autoantibody-positive result showed an increase in score of approximately 15 points for state anxiety with a very large effect size (*d*=2.19), indicating a pronounced acute stress response to risk disclosure; trait anxiety also increased (*d*=0.69), suggesting effects beyond a transient fluctuation.

Taken together with the reductions in anxiety symptoms among parents of autoantibody-negative children, these findings indicate that the psychological impact of screening diverges substantially by test outcome. Accordingly, presymptomatic type 1 diabetes screening should pair immunological testing with outcome-specific, two-step risk communication and structured counselling that accounts for the distinct psychological burden of positive vs negative results [19].

### Clinical and public health implications

Given Turkey’s high DKA rates at diagnosis [5–7], presymptomatic screening should be considered a public health priority. Large-scale programmes show that such screening reduces DKA and is cost-effective [9–12]. The higher frequency of ZnT8A positivity in early childhood suggests that those at greatest DKA risk may be identifiable earlier through targeted screening. These findings support the value of both targeted and population-based approaches, particularly in younger age groups, to improve outcomes and optimise healthcare resources.

Advances in immune-directed therapies, including Treg-based approaches centred on the FOXP3 axis [41, 42], such as polyclonally expanded Tregs and CAR-Treg strategies [43, 44], further strengthen the rationale for presymptomatic identification as an opportunity for immune modulation before irreversible beta cell loss. Integrating biological and psychosocial components, including structured result-specific counselling, is essential for programme effectiveness and sustainability, consistent with ISPAD guidance on family-centred screening and follow-up [13].

### Limitations

Limitations include the cross-sectional design, which precludes causal inference and determination of longitudinal autoantibody trajectories, and the single post-disclosure assessment of anxiety symptoms, which may capture transient fluctuations rather than long-term effects. Anxiety in children was assessed using SCARED, whereas parental anxiety was assessed using STAI-I/II. These instruments do not serve identical purposes: SCARED is a multidimensional symptom-screening tool that is more reflective of broader anxiety symptom burden, whereas STAI-I is designed to assess state anxiety and acute emotional responses to a stressor at a given moment. Therefore, the child and parent anxiety findings should not be interpreted as measuring the exact same psychological construct. In addition, although SCARED provides a reliable basis for anxiety assessment across a broad paediatric age range, it is a general symptom-screening instrument rather than a dedicated state-anxiety measure. Therefore, its sensitivity to short-term anxiety fluctuations related to islet autoantibody result disclosure may be limited. In addition, selection bias related to volunteer participation, multiple comparisons and residual confounding cannot be excluded, and low-titre assay variability may have introduced some misclassification. Generalisability is constrained by the nine-centre design within Turkey, the sibling-dominant cohort structure, and the limited number of perinatal variables assessed. Nonetheless, the results suggest that presymptomatic type 1 diabetes screening should pair testing with structured, stepwise psychosocial support.

### Conclusion

To our knowledge, this is the first multicentre screening study from Turkey among paediatric FDRs of individuals with type 1 diabetes to address both biological and anxiety symptom dimensions, providing a basis for national screening strategies. The present findings should be interpreted primarily within the context of a sibling-dominant paediatric FDR cohort. ZnT8A was the predominant islet autoantibody, and its presence was inversely associated with age, supporting its value as an early-stage biomarker. Gestational age was an independent protective factor, with each additional week of gestation associated with an approximately 20% lower odds of autoantibody positivity, highlighting the contribution of perinatal biology to type 1 diabetes risk stratification. The marked increase in anxiety symptoms in both children and parents after disclosure of a positive result suggests that presymptomatic screening should integrate psychological safety measures and structured counselling alongside immunological assessment. Future prospective studies should assess the long-term impact of ZnT8A positivity and gestational factors on disease progression, and randomised trials should test brief interventions to mitigate stress related to risk disclosure.

## Supplementary Information

Below is the link to the electronic supplementary material.

ESM (PDF 303 KB)

## Data Availability

The datasets generated and analysed during the current study are not publicly available because they contain participant-level clinical and psychometric data from minors. De-identified data underlying the findings may be made available from the corresponding author upon reasonable request after publication and for 5 years thereafter, for non-commercial academic research with a methodologically sound proposal, subject to approval by the study investigators, and, where required, the relevant ethics committee and/or institutional data-sharing procedures. Data may not be used to identify participants or shared with third parties.

## Abbreviations

aOR: Adjusted OR
DKA: Diabetic ketoacidosis
FDR: First-degree relative
SCARED: Screen for Child Anxiety Related Emotional Disorders
STAI-I/II: State–Trait Anxiety Inventory I/II
