# Supplementary material for: Autoantibody screening in children who are first-degree relatives of individuals with type 1 diabetes in a sibling-dominant cohort in Turkey: a multicentre study on prevalence, determinants and post-screening parent–child anxiety
Source: Diabetologia. 2026 Jun 25;69(9):2432–44. doi: 10.1007/s00125-026-06783-6 (PMC13424225; doi:10.1007/s00125-026-06783-6)
Supplement: Supplementary file 1 — ESM (PDF 303 KB) [file 125_2026_6783_MOESM1_ESM.pdf]

Electronic supplementary material (ESM)

ESM Table 1. Demographic and descriptive characteristics of participants with complete paired anxiety assessments (*N*=192).

**Characteristic**	***n* (%) / mean ± SD (range)**
Sex	
Female	108 (56)
Male	84 (44)
Autoantibody status	
Positive	26 (14)
Negative	166 (86)
Age (years)	12.32 ± 2.99 (8 - 18)

Notes: This table summarizes participants with complete paired pre-disclosure and post-disclosure anxiety assessments (children: SCARED; parents: STAI-I/II). Data are presented as *n* (%) or mean ± SD (range). Autoantibody-positive indicates >=1 islet autoantibody. Percentages are calculated within *N*=192 and may not sum to 100 due to rounding. Abbreviations: SCARED, Screen for Child Anxiety Related Emotional Disorders; STAI, State-Trait Anxiety Inventory.

ESM Table 2. Correlation between age and change in SCARED total score (ΔSCARED).

	Total sample (N=192) (r / p)	***Female (N=108) (r / p)***	***Male (N=84) (r / p)***
Age - ΔSCARED	0.012 / 0.872	0.044 / 0.648	-0.031 / 0.779

Notes: DeltaSCARED was calculated as post-disclosure minus pre-disclosure total SCARED score; positive values indicate increased anxiety after disclosure/counseling. Pearson correlation coefficients (r) with two-sided *p* values are shown for the total sample and stratified by sex. Abbreviations: r, correlation coefficient; SCARED, Screen for Child Anxiety Related Emotional Disorders.

ESM Table 3. Pre-disclosure and post-disclosure SCARED total and subscale scores in autoantibody-positive children (*n* = 26)

**SCARED scale**	**Pre-disclosure (mean ± SD)**	**Post-disclosure (mean ± SD)**	***p***	**Cohen's *d***
SCARED total	20.15 ± 10.47	32.88 ± 13.83	**<0.001**	1.32
Panic/somatic symptoms	4.27 ± 2.18	14.12 ± 5.47	**<0.001**	2.09
Generalised anxiety	3.27 ± 1.69	10.27 ± 4.07	**<0.001**	2.07
Separation anxiety	5.04 ± 2.97	3.42 ± 2.32	**<0.001**	0.77
Social anxiety	4.19 ± 2.38	2.92 ± 1.83	0.001	0.71
School avoidance	3.38 ± 1.86	2.15 ± 1.46	**<0.001**	0.85

Pre-disclosure and post-disclosure SCARED total and subscale scores were compared using paired Student's t tests (n = 26 autoantibody-positive children). Because five separate subscale comparisons were performed, the Bonferroni-corrected significance threshold was set at p < 0.010. Cohen's d effect sizes are reported as absolute values.

SCARED, Screen for Child Anxiety Related Emotional Disorders; SD, standard deviation

ESM Table 4. Pre-disclosure and post-disclosure SCARED total and subscale scores in autoantibody-negative children (*n* = 166)

**SCARED scale**	**Pre-disclosure (mean ± SD)**	**Post-disclosure (mean ± SD)**	***p***	**Cohen's *d***
SCARED total	23.88 ± 12.43	17.85 ± 8.72	**<0.001**	0.62
Panic/somatic symptoms	5.73 ± 3.25	4.95 ± 3.06	0.010	0.20
Generalised anxiety	4.57 ± 2.77	3.90 ± 2.22	0.005	0.22
Separation anxiety	5.43 ± 3.05	3.58 ± 2.28	**<0.001**	0.63
Social anxiety	4.55 ± 2.57	3.04 ± 1.88	**<0.001**	0.61
School avoidance	3.54 ± 1.81	2.28 ± 1.76	**<0.001**	0.75

Pre-disclosure and post-disclosure SCARED total and subscale scores were compared using paired Student's t tests (n = 166 autoantibody-negative children). Because five separate subscale comparisons were performed, the Bonferroni-corrected significance threshold was set at p < 0.010. Cohen's d effect sizes are reported as absolute values.

SCARED, Screen for Child Anxiety Related Emotional Disorders; SD, standard deviation

ESM Table 5. Multiple linear regression analysis of factors predicting change in SCARED total score (ΔSCARED) in children and adolescents.

Predictor	B	SE	β	*t*	*p*	Tolerance	VIF
Autoantibody positivity (0=negative, 1=positive)	16.868	1.581	0.499	10.67	***p* < 0.001**	0.989	1.011
SCARED (pre-disclosure)	-0.508	0.044	-0.535	-11.45	***p* < 0.001**	0.989	1.011

Notes: *The dependent variable was ΔSCARED, defined as post-disclosure minus pre-disclosure total SCARED score; positive values indicate increased anxiety after disclosure, whereas negative values indicate decreased anxiety. Autoantibody positivity was coded as 0 = negative and 1 = positive. B indicates the unstandardised regression coefficient; SE, standard error; β, standardised coefficient. Tolerance and VIF values assess multicollinearity. Two-sided p values are shown; statistical significance was set at p < 0.01. Abbreviations: VIF, variance inflation factor.*

ESM Table 6. Mixed-design ANOVA results for anxiety levels in the autoantibody-negative group

**Effect**	**SS**	***df***	**MS**	***F***	***p***	**η^2_p**
Within-subjects effects						
Time (pre–post)	3007.53	1	3007.53	63.66	**<0.001**	0.282
Time × Child sex	41.01	1	41.01	0.87	0.353	0.005
Time × Sibling sex	24.36	1	24.36	0.52	0.474	0.003
Time × Child sex × Sibling sex	1.08	1	1.08	0.02	0.880	0.000
Error (within)	7654.10	162	47.25			
Between-subjects effects						
Child sex	1.67	1	1.67	0.009	0.925	0.000
Sibling sex	74.39	1	74.39	0.399	0.529	0.002
Child sex × Sibling sex	13.71	1	13.71	0.073	0.787	0.000
Error (between)	30238.79	162	186.66			

Analyses were conducted only in the autoantibody-negative group (*n*=166). The time factor represents pre-disclosure and post-disclosure measurements of child anxiety (SCARED total score). Child sex and sibling sex refer to the sex of the screened child and the sex of their sibling with T1D, respectively

df, degrees of freedom; MS, mean square; SCARED, Screen for Child Anxiety Related Disorders; SS, sum of squares; T1D, type 1 diabetes; η^2_p, partial eta squared

ESM Table 7. Comprehensive comparison of parental state and trait anxiety scores: total sample and subgroups

**Group/Subgroup**	**Measure**	**Mean ± SD**	***t* (df)**	***p***	**Cohen's *d***
**Total Sample (*N*=192)**	State Anxiety (Pre)	35.10 ± 8.60			
	State Anxiety (Post)	32.22 ± 8.89			
	ΔState Anxiety	−2.88 ± 10.27	−3.89 (191)	**<0.001****	0.28
	Trait Anxiety (Pre)	36.67 ± 8.45			
	Trait Anxiety (Post)	34.83 ± 8.33			
	ΔTrait Anxiety	−1.83 ± 7.16	−3.55 (191)	**<0.001****	0.26
By Autoantibody Status					
Autoantibody-Positive (*n*=26)	State Anxiety (Pre)	30.67 ± 8.18			
	State Anxiety (Post)	45.78 ± 7.46			
	ΔState Anxiety	+15.11 ± 6.89	11.39 (25)	**<0.001****	2.19
	Trait Anxiety (Pre)	31.26 ± 5.91			
	Trait Anxiety (Post)	37.19 ± 8.84			
	ΔTrait Anxiety	+5.93 ± 8.59	3.58 (25)	**0.001****	0.69
Autoantibody-Negative (*n*=166)	State Anxiety (Pre)	35.83 ± 8.47			
	State Anxiety (Post)	30.01 ± 6.94			
	ΔState Anxiety	−5.82 ± 7.31	−10.24 (165)	**<0.001****	0.80
	Trait Anxiety (Pre)	37.55 ± 8.49			
	Trait Anxiety (Post)	34.45 ± 8.20			
	ΔTrait Anxiety	−3.10 ± 6.04	−6.60 (165)	**<0.001****	0.51

Abbreviations: *SD, standard deviation; Δ, change (post-disclosure minus pre-disclosure); STAI, State–Trait Anxiety Inventory.*
Notes: *State anxiety (STAI-I) reflects situational anxiety at the time of assessment; trait anxiety (STAI-II) reflects general anxiety proneness. ΔState Anxiety and ΔTrait Anxiety were calculated as post-disclosure minus pre-disclosure scores; positive values indicate increased anxiety after disclosure, whereas negative values indicate decreased anxiety. p values are from paired Student's t tests comparing pre-disclosure and post-disclosure scores within each group.*

ESM Fig. 1. Psychological effect sizes by autoantibody status.

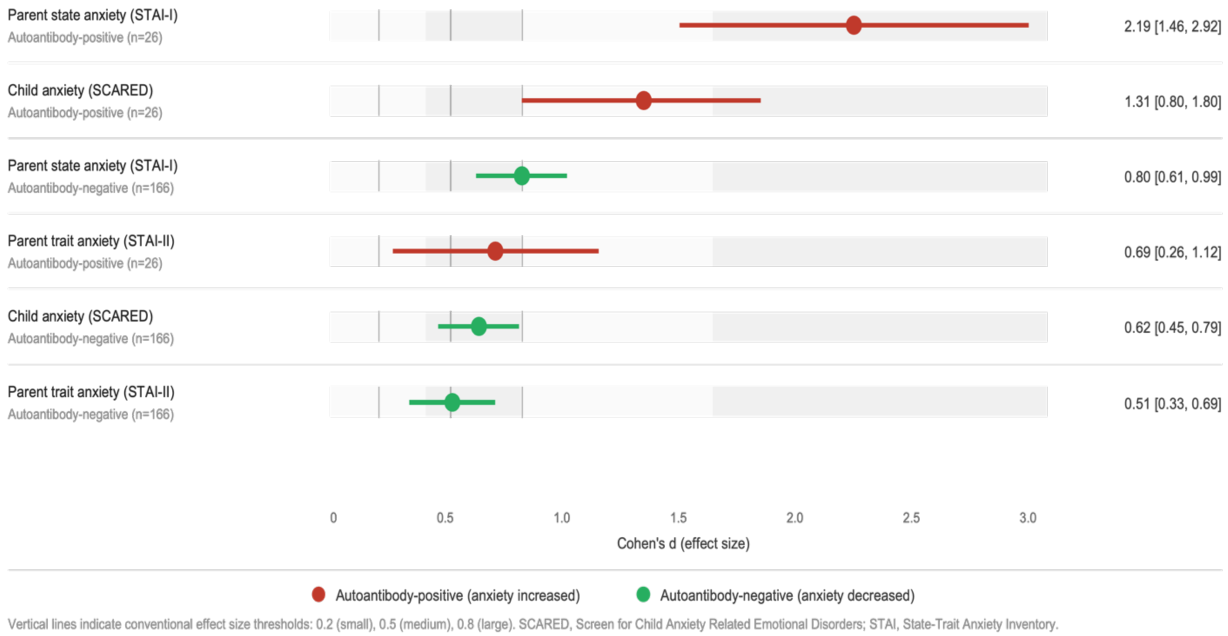

Forest plot displaying Cohen's *d* (points) with 95% confidence intervals (horizontal bars) for within-group changes from pre- to post-disclosure of islet autoantibody screening results in parent state anxiety (STAI-I), parent trait anxiety (STAI-II), and child anxiety symptoms (SCARED). Effect sizes are shown separately for families of autoantibody-positive children (Ab+, *n*=26) and autoantibody-negative children (Ab−, *n*=166). Red markers indicate increased anxiety after disclosure, whereas green markers indicate decreased anxiety. Vertical reference lines denote conventional effect size thresholds (*d* = 0.2 small, 0.5 medium, 0.8 large).

Abbreviations: Ab+, autoantibody-positive; Ab−, autoantibody-negative; SCARED, Screen for Child Anxiety Related Emotional Disorders; STAI, State–Trait Anxiety Inventory (STAI-I, State; STAI-II, Trait).
